# Ventilation distribution as a contributor to the functional exercise capacity in patients with systemic sclerosis-associated interstitial lung disease without pulmonary hypertension

**DOI:** 10.1590/1414-431X20198513

**Published:** 2019-07-29

**Authors:** F.M. Andrade, A.D. Oliveira, A.J. Lopes

**Affiliations:** 1 Programa de Pós-Graduação em Ciências da Reabilitação, Centro Universitário Augusto Motta, Rio de Janeiro, RJ, Brasil; 2 Programa de Pós-Graduação em Ciências Médicas, Faculdade de Ciências Médicas, Universidade do Estado do Rio de Janeiro, Rio de Janeiro, RJ, Brasil

**Keywords:** Systemic sclerosis, Respiratory function tests, Ventilation, Nitrogen single-breath washout test, Exercise, Six-minute walk test

## Abstract

Phenotypic differences have been described between patients with systemic sclerosis (SSc)-associated interstitial lung disease (ILD) and SSc-associated pulmonary hypertension, including performance differences in the 6-min walk test (6MWT). Moreover, the correlations between the 6MWT and traditional pulmonary function tests (PFTs) are weak, indicating the need to search for new parameters that explain exercise performance. Thus, our objective was to evaluate the impact of ventilation distribution heterogeneity assessed by the nitrogen single-breath washout (N_2_SBW) test and peripheral muscle dysfunction on the exercise capacity in patients with SSc-ILD and limited involvement of the pulmonary parenchyma. In this cross-sectional study, 20 women with SSc-ILD and 20 matched controls underwent PFTs (including spirometry, diffusing capacity for carbon monoxide (DLco), and the N_2_SBW test) and performed the 6MWT and knee isometric dynamometry. The 6-min walking distance (6MWD, % predicted) was strongly correlated with the phase III slope of the single-breath nitrogen washout (phase III slope_N2SBW_) (r=−0.753, P<0.0001) and reasonably correlated with the forced vital capacity (FVC) (r=0.466, P=0.008) and DLco (r=0.398, P=0.011). The peripheral oxygen saturation (SpO_2_) during exercise was not significantly correlated with any of the pulmonary or muscle function parameters. The phase III slope_N2SBW_ was the only predictive variable for the 6MWD, whereas quadriceps strength and FVC/DLco were predictive variables for SpO_2_. Ventilation distribution heterogeneity is one factor that contributes to a lower 6MWD in SSc-ILD patients. In addition, muscle dysfunction and abnormal lung diffusion at least partly explain the decreased SpO_2_ of these patients.

## Introduction

Recently, important differences among the various clinical phenotypes of systemic sclerosis (SSc) have been increasingly identified, which has made comparisons among patients within the same group difficult. Regarding cardiopulmonary involvement, large differences in phenotypic expression have been found between subjects with SSc-associated interstitial lung disease (SSc-ILD) and SSc-associated pulmonary hypertension (SSc-PH) (1,2). In SSc-ILD, the excessive presence of collagen causes irreversible damage to the pulmonary structures, alveolar walls, and interstitial spaces, which alter respiratory mechanics and gas exchange (3).

In current clinical practice, lung damage severity is most frequently quantified and monitored using pulmonary function tests (PFTs) (4). Although forced vital capacity (FVC) is the most frequently used primary endpoint in clinical trials, there is little evidence to support its superiority over other PFTs, and rigorous validation of its performance characteristics is lacking (5,6). Moreover, the high signal-to-noise ratio of the FVC trajectory is a significant problem that makes this measurement an unreliable tool for predicting results at the individual patient level (2). These limitations raise questions regarding alternative measures that may facilitate clinical decision-making in SSc-ILD cases. Recently, interest has been increasing in the use of the nitrogen single-breath washout (N_2_SBW) test for early diagnosis of small airway involvement and stratification of lung damage severity in various systemic diseases (7–9). From the pathophysiological perspective, the N_2_SBW test has been used to measure homogeneity in the ventilation distribution for various clinical conditions (7). A recent study used the N_2_SBW test in SSc patients. The results showed that heterogeneity in the ventilation distribution was a frequent finding in these patients and that this change was found even in the absence of restrictive damage based on PFTs (10).

Similar to cardiopulmonary involvement, skeletal muscle involvement in SSc may be an early indicator of an unfavorable prognosis (11). Due to the different criteria used to define muscle involvement in SSc, its real prevalence is not clearly established and has a range of 5–96% (11,12). Although muscle weakness may be associated with myopathy, it may also be secondary to disuse due to skin thickening and joint contracture (13). In addition, the drugs used to treat SSc-ILD, including glucocorticoids and immunobiological drugs, may contribute to reduced muscle performance (14). However, the impact of peripheral muscle dysfunction on poor performance during exercise has been poorly explored in SSc-ILD patients.

The 6-min walk test (6MWT) is a simple submaximal exercise test that is safe, noninvasive, reproducible, and reliable, and correlates with daily physical activity (15). Unfortunately, in general, SSc patients not only suffer from cardiopulmonary or musculoskeletal disease but also exhibit combinations of cardiac involvement, lung injury, skin fibrosis, muscle damage, and joint disease that may confound interpretation of the 6MWT results (16). Despite this challenge, the 6MWT has been increasingly used to evaluate performance in physical exercise and as a tool for follow-up, and a primary measure of therapy outcomes and responses (16). Currently, the 6MWT combined with measurements of the diffusing capacity for carbon monoxide (DLco) is used to identify SSc-ILD patients at a higher risk of developing SSc-PH (17), because important differences exist among the performances of SSc-ILD, SSc-PH, and SSc-ILD-PH patients in the 6MWT (16).

Although the 6MWT is highly reproducible in SSc-ILD patients, its value has been questioned because of poor correlations between the distance walked and clinical parameters, lung function measures (including FVC and DLco), and the disease extent on high-resolution computed tomography (HRCT) (5,17,18). Therefore, there is a need to seek better parameters in PFTs that may explain the functional disability in exercise in SSc-ILD patients. With increasing incorporation of the N_2_SBW test in clinical practice, we hypothesized that the parameters extracted from this test might help explain the reduced functional capacity in this patient population. Thus, our objective was to evaluate the impact of ventilation distribution heterogeneity assessed by the N_2_SBW test and peripheral muscle dysfunction on the functional capacity during exercise in SSc-ILD patients with limited pulmonary parenchymal involvement.

## Material and Methods

### Patients

Between March and November 2018, a cross-sectional study was conducted on 32 women aged ≥18 years who were diagnosed with SSc-ILD based on HRCT. These patients were regularly seen at Hospital Universitário Pedro Ernesto, Rio de Janeiro, Brazil and were diagnosed with the disease according to the American College of Rheumatology Classification Criteria (19). Only patients whose HRCT examinations showed limited ILD (i.e., lung parenchymal involvement <20%) were included (20). The exclusion criteria were as follows: evidence of overlap with other connective tissue diseases; patients with SSc-PH, including PH due to vasculopathy of the small pulmonary arteries (group 1, SSc-associated pulmonary arterial hypertension (SSc-PAH), ILD (group 3, PH due to pulmonary disease or chronic hypoxia), or left ventricular dysfunction (group 2, PH due to chronic left heart disease) (21); a smoking history ≥10 pack-years; a history of unstable angina or heart attack during the previous month; a report of infection within the past 4 weeks; neurological or orthopedic disorders; pain in the lower extremities; joint deformities causing movement limitations; or any other deficiency that would make the individuals incapable of performing the 6MWT. Regarding cutaneous involvement, the patients were classified as having the limited (lc-SSc) or diffuse (dc-SSc) form according to the classification (22). The modified Rodnan skin score (mRSS) was used to assess skin damage; in this system, a score of 0 (no thickening), 1 (light thickening), 2 (moderate thickening), or 3 (severe thickening) is assigned at 17 different body sites, resulting in a total score ranging from 0 to 51 (23). The disease duration was defined as the time since the onset of the first symptom, except for Raynaud's phenomenon (RP).

A control group of 20 healthy women aged ≥18 years was recruited at Centro Universitário Augusto Motta (UNISUAM), Brazil. These women did not report smoking ≥10 pack-years or a history of cardiorespiratory or musculoskeletal disorders.

The entire protocol followed the recommendations for research on humans according to the 1964 Helsinki Declaration and its subsequent revisions. The protocol was approved by the Research Ethics Committee of UNISUAM under CAAE No. 77203417.1.0000.5235, and all patients signed the consent form.

### Measurements

#### Lung function

The spirometry and DLco measurement were performed with the Collins Plus Pulmonary Function Testing System (Warren E. Collins, Inc., USA) according to the recommendations of the American Thoracic Society/European Respiratory Society (24). The reference values were provided by Pereira et al. (25) for spirometry and by Neder et al. (26) for DLco, and the results are reported as percent predicted values. In addition, we performed the N_2_SBW test on the HDpft 3000 system (nSpire Health, Inc., USA) following previously established recommendations (7). Two parameters derived from the test were reported relative to the predicted values of Teculescu et al. (27): the phase III slope of the nitrogen single-breath washout (phase III slope_N2SBW_), which is a change in the N_2_ concentration between 25 and 75% of the expiratory volume; and the closing volume/vital capacity (CV/VC), which is the portion of the VC that is exhaled after the start of airway closure.

#### Knee isometric dynamometry

The muscle strength of the lower limbs was evaluated using an isometric dynamometer (model DIN-TRO, EMG System do Brasil Ltda., Brazil), and the endurance test was performed using a surface electromyography device (EMG model 810C, EMG System do Brasil LTDA.). Participants were instructed to cross their arms over their chest while the seat was adjusted to 90° of hip flexion. Surface electrodes were placed on the quadriceps according to previous recommendations (28). Maximal isometric voluntary contraction (MIVC) was performed at the knee for the quadriceps muscles with leg extensions. Each test was performed 3 times, and the highest value was selected. The endurance evaluation consisted of a sustained 60-s contraction using 30% of the MIVC obtained in the strength test. The median frequency and root mean square slope (MDF slope and RMS slope, respectively) were used to analyze quadriceps fatigue (28).

#### Six-minute walk test

The 6MWT was conducted in a long, flat, straight, and closed corridor with a 30-m walking track. All participants were previously familiarized with the procedure. The heart rate, respiratory rate, blood pressure, peripheral oxygen saturation (SpO_2_), and Borg dyspnea index were measured before and at the end of the test following the recommendations of the American Thoracic Society (15). Oxygen desaturation was defined as a decrease in SpO_2_ ≥4% (severe desaturation, SpO_2_ ≤88%) at the end of the test (29). The percentage predicted values were calculated using previously published equations (30).

### Statistical analysis

Nonparametric methods were applied, because the variables did not present a Gaussian distribution according to rejection of data normality by the Shapiro-Wilk test. The variables were compared between patient groups and healthy controls using the Mann-Whitney test. The Spearman correlation coefficient (r_s_) was used to evaluate associations between variables. Correlations between 0 and 0.25 (or 0 and −0.25) were considered small or nonexistent, between 0.26 and 0.50 (or −0.26 to −0.50) were considered reasonable, between 0.51 and 0.75 (or −0.51 to −0.75) were considered moderate to good, and greater than 0.75 (or -0.75) were considered very good to excellent (31). In addition, multiple linear regression analysis was applied to identify pulmonary function and muscle function variables that were independent for prediction of 6MWT variables after considering confounding factors (including clinical and demographic data). The backward stepwise method was performed to select independent variables in the multiple linear regression models, and only variables with P<0.10 in the bivariate analysis were retained in the final model. Due to the lack of normality in the 6-min walking distance (6MWD) and SpO_2_ distributions, logarithmic transformation (natural log) was applied to fit the regression models appropriately.

The data analysis was performed using SAS 6.11 (SAS Institute, Inc., USA). The results are reported as the medians and interquartile ranges or as frequencies (percentages), and statistical significance was considered when P<0.05.

## Results

Among the 32 patients included in the study, 12 were excluded for the following reasons: 6 presented SSc-PH; 3 showed overlap with other connective tissue diseases; 2 reported a smoking history ≥10 pack-years; and 1 because of joint deformities that impaired the 6MWT performance. Thus, the sample consisted of 20 women with SSc-ILD with a limited disease extent based on HRCT. The median age was 51 (40.5−59.8) years. Eleven patients had lc-SSc and 9 had dc-SSc according to a previously published classification (22). The median mRSS was 10.5 (4.18–16.2). Regarding use of medication, 11 used corticosteroids, 14 used immunosuppressants, and 4 used immunobiological drugs. The median disease duration was 4 (2–9.50) years. Regarding lung function, 10 patients had an FVC <80% predicted, 12 had a DLco <80% predicted, and 1 had an FVC/DLco >1.6. In the N_2_SBW test, the phase III slope_N2SBW_ and CV/VC ratio were above 120% predicted in 15 and 7 patients, respectively. Regarding the 6MWT, the median distance walked was 417.5 (345–491.5) meters and was <80% of that predicted in 11 cases. The median SpO_2_ values before and after the 6MWT were 98% (99–96) and 97% (98–93), respectively, and 5 patients had desaturations at the end of the 6MWT (3 had severe desaturations).

Table 1 provides the demographic data, lung function, physical function, peripheral muscle performance, and functional capacity of the control subjects and SSc-ILD patients. In the PFTs, the patients had lower FVC (77.5 (56.8−95) *vs* 99.5 (88.3−108) % predicted, P=0.0001) and DLco values (77 (47.8−110) *vs* 104.5 (90−111) % predicted, P=0.013) and higher phase III slope_N2SBW_ values (229 (106−455) *vs* 105 (94−112) % predicted, P=0.004) than the controls. In the 6MWT, the patients had lower 6MWD values (77.5 (56.8−95) *vs* 99.5 (88.3−108) % predicted, P=0.006) and higher SpO_2_ drops (1 (0–1) *vs* 2 (1–4) % predicted, P=0.032). No significant differences were observed between the patients and controls with respect to the parameters provided by knee isometric dynamometry.

**Table 1.** Demographic data, lung function, physical function, peripheral muscle performance, and functional capacity of control subjects and systemic sclerosis-associated interstitial lung disease patients (SSc-ILD).

Variable	Control subjects (n = 20)	SSc-ILD patients (n = 20)	P value
Demographic data			
Age (years)	57.5 (29.3–68.8)	51 (40.5–59.8)	0.54
Weight (kg)	64.5 (57.5–69.5)	71 (53.5–83)	0.24
Height (cm)	159 (151–166)	161 (154–164)	0.65
BMI (kg/m^2^)	25.5 (21.4–28.9)	25.9 (21.4–32.3)	0.43
Lung function			
FVC (% predicted)	99.5 (88.3–108)	77.5 (56.8–95)	0.0001
DLco (% predicted)	104.5 (90–111)	77 (47.8–110)	0.013
FVC/DLco (%)	0.97 (0.86–1.10)	1.06 (0.76–1.25)	0.47
Phase III slope_N2SBW_ (% predicted)	105 (94–112)	229 (106–455)	0.004
CV/VC (% predicted)	85 (75.8–111)	93.5 (63.3–143)	0.64
Peripheral muscle performance			
RMS slope	0.08 (-0.12–0.24)	0.10 (-0.35–0.77)	0.55
MDF slope	0.07 (0.01–0.39)	0.02 (-0.03–-0.18)	0.10
Quadriceps strength (kg)	34.2 (21.6–39.9)	30.1 (16.4–36.7)	0.40
6-min walk test			
6MWD (m)	462 (393.5–541)	417.5 (345–491.5)	0.012
6MWD (% predicted)	85.2 (81.9–92.6)	76.7 (68.5–86.8)	0.006
ΔSpO_2_ (% pre-post 6MWD)	1 (0–1)	2 (1–4)	0.032

Data are reported as medians and interquartile ranges. BMI: body mass index; FVC: forced vital capacity; DLco: diffusing capacity for carbon monoxide; Phase III slope_N2SBW_: phase III slope of the nitrogen single-breath washout; CV/VC: closing volume/vital capacity; RMS: angle of the linear regression line obtained with the values of the root mean square electromyography signal over time during the fatigability test of the vastus medialis muscle; MDF: angle of the linear regression line obtained with the values of the median frequency of the electromyography signal over time during the fatigability test of the vastus medialis muscle; 6MWD: 6-min walking distance; ΔSpO_2_: peripheral oxygen saturation.

**Figure 1.**
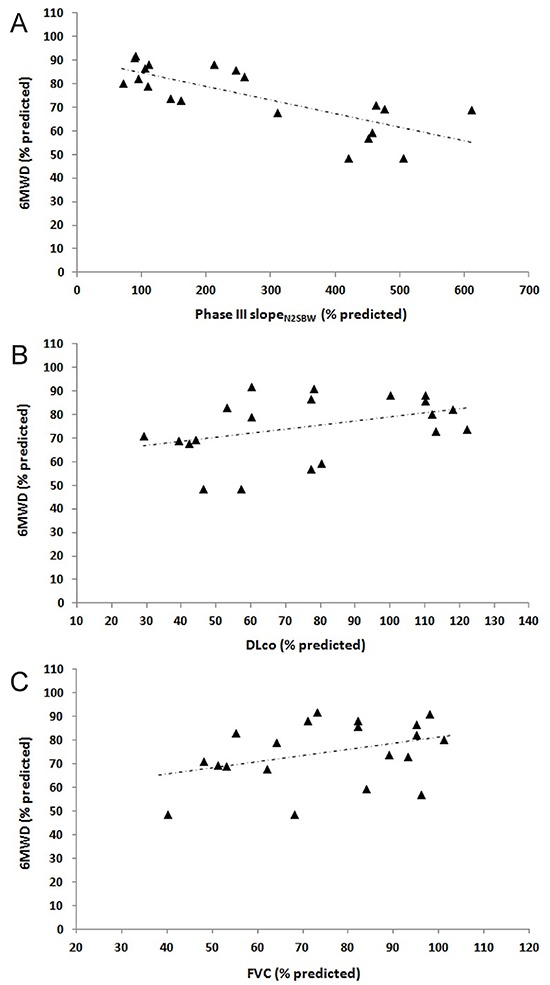
**A**, Relationship of the 6-min walking distance (6MWD, % predicted) with the phase III slope of the nitrogen single-breath washout (phase III slope_N2SBW_) (r=−0.753, P<0.0001), **B**, diffusing capacity for carbon monoxide (DLco) (r=0.398, P=0.011), and **C**, forced vital capacity (FVC) (r=0.466, P=0.008).

Table 2 and Figure 1 show the correlations of the 6MWD (% predicted and meters) and ΔSpO_2_ with lung function and peripheral muscle parameters. In this analysis, the 6MWD (% predicted) was strongly correlated with the phase III slope_N2SBW_ (r=-0.753, P<0.0001) and reasonably correlated with the FVC (r=0.466, P=0.008) and DLco (r=0.398, P=0.011). The 6MWD (meters) was moderately correlated with the phase III slope_N2SBW_ (r=−0.594, P=0.0006) and reasonably correlated with the FVC (r=0.412, P=0.010) and DLco (r=0.363, P=0.025). SpO_2_ was not significantly correlated with any of the pulmonary or muscle function parameters. The correlations of the 6MWD with the ΔSpO_2_ and mRSS were not significant (r=−0.236, P=0.32 and r=−0.250, P=0.27, respectively).

**Table 2.** Spearman's correlation coefficients for the 6-min walking distance and peripheral oxygen saturation with lung function and peripheral muscle parameters.

Variables	6MWD (% predicted)		6MWD (meters)		ΔSpO_2_ (% pre-post 6MWD)	
	r	P value	r	P value	r	P value
FVC (% predicted)	0.466	0.008	0.412	0.010	–0.026	0.91
DLco (% predicted)	0.398	0.011	0.363	0.025	–0.234	0.32
FVC/DLco (%)	–0.239	0.31	–0.220	0.35	0.291	0.09
Phase III slope_N2SBW_ (% predicted)	–0.753	<0.0001	–0.594	0.0006	0.062	0.80
CV/VC (% predicted)	–0.300	0.20	–0.266	0.26	0.051	0.83
RMS slope	–0.180	0.45	–0.105	0.66	–0.033	0.89
MDF slope	0.205	0.39	0.138	0.56	–0.227	0.33
Quadriceps strength (kg)	0.280	0.23	0.191	0.42	–0.323	0.08

6MWD: 6-min walking distance; ΔSpO_2_: peripheral oxygen saturation; FVC: forced vital capacity; DLco: diffusing capacity for carbon monoxide; Phase III slope_N2SBW_: phase III slope of the nitrogen single-breath washout; CV/VC: closing volume/vital capacity; RMS: angle of the linear regression line obtained with the values of the root mean square electromyography signal over time during the fatigability test of the vastus medialis muscle; MDF: angle of the linear regression line obtained with the values of the median frequency electromyography signal over time during the fatigability test of the vastus medialis muscle.

We also evaluated the associations of the FVC and DLco values (% predicted) with the N_2_SBW test and knee isometric dynamometry parameters. The values of the correlations with the FVC were as follows: phase III slope_N2SBW_ (r=−0.761, P<0.0001); CV/VC (r=−0.387, P=0.012); RMS slope (r=−0.045, P=0.85); MDF slope (r=0.110, P=0.64); and quadriceps strength (r=0.138; P=0.56). The values of the correlations with the DLco were as follows: phase III slope_N2SBW_ (r=−0.372, P=0.017); CV/VC (r=−0.261, P=0.27); RMS slope (r=−0.023, P=0.92); MDF slope (r=0.124, P=0.62); and quadriceps strength (r=0.065; P=0.78).

Finally, we investigated whether the pulmonary and muscle function variables could independently predict performance during the 6MWT (% predicted) and the ΔSpO_2_ (Table 3). In this analysis, the phase III slope_N2SBW_ was the only independent predictive variable for the 6MWD, whereas quadriceps strength and the FVC/DLco ratio were independent predictive variables for ΔSpO_2_.

**Table 3.** Independent linear models for variables of the 6-min walk test using lung function and peripheral muscle parameters.

Outcome variables	Independent variables	B	SEB	P value	Cumulative R^2^
6MWD	Phase III slope_N2SBW_	–0.0006	0.0001	0.0001	0.60
ΔSpO_2_	Quadriceps strength	–0.082	0.025	0.022	0.14
	FVC/DLco	2.475	1.178	0.043	0.26

B: regression coefficient; SEB: standard error of the regression coefficient; R^2^: determination coefficient; 6MWD: 6-min walking distance; Phase III slope_N2SBW_: phase III slope of the nitrogen single-breath washout; ΔSpO_2_: peripheral oxygen saturation; FVC: forced vital capacity; DLco: diffusing capacity for carbon monoxide.

## Discussion

In this study, we aimed to assess the impact of ventilation distribution heterogeneity and peripheral muscle function on the 6MWT performance in SSc-ILD patients with limited pulmonary parenchymal involvement and without PH. The choice of this patient subgroup was justified by differences in the phenotypic expression of SSc (1,2,17), which increases the difficulty of interpreting the results of previous studies that evaluated patients in a single group. This problem has also been highlighted recently with regard to the 6MWT, because SSc-PAH patients walk less than SSc-non-PAH patients and SSc-ILD-PH patients walk less than SSc-ILD and SSc-non-ILD patients (16). Accordingly, we also excluded patients with any condition unrelated to SSc that could impact peripheral muscle function and, consequently, exercise performance. Notably, our sample was comprised exclusively of women, and this composition was in accordance with the gender distribution reported for SSc indicating a majority of female cases (32).

Similar to the study by Silva et al. (10), we observed that an increased phase III slope_N2SBW_, which is an indicator of inhomogeneity in ventilation distribution, was the most common change in lung function, suggesting the potential of this index as a marker of lung damage in SSc-ILD patients. High phase III slope_N2SBW_ values indicate a poor ventilation distribution, because this variable measures regional differences in respiratory system time constants due to changes in airway resistance and lung distensibility, which in turn compromise alveolar emptying. In SSc-ILD, involvement of the pulmonary interstitium with structural derangement due to excessive collagen secretion may cause a poor ventilation distribution (13). This situation is relevant even in patients with mild ILD, which is characteristic of our sample, in whom the HRCT showed lung parenchymal involvement of <20%. Interestingly, Guler et al. (2) showed that SSc-ILD patients have distinct physiological progression patterns that remain relatively consistent during long-term follow-up and that the rate of decline in FVC has limited value in predicting future disease progression. Thus, we think that clinical trials can be designed to evaluate longitudinal changes in the phase III slope_N2SBW_ in SSc-ILD patients to validate the applicability of this measure for disease monitoring.

In addition to the myopathy in SSc patients, chronic hypoxemia resulting from pulmonary disease can contribute to inflammation modulation and therefore damage type II muscle fibers, negatively affecting the performance of the peripheral muscles (33). However, we did not observe significant differences between the patients and healthy controls in lower limb strength and endurance measures in the present study. These findings are different from those observed by Lima et al. (14), who used the same measurement instrument as our study (knee isometric dynamometry) and demonstrated a reduction in strength and increase in quadriceps fatigability in SSc patients. The differences in the results between the two studies might be partially explained by differences in the populations studied, because we excluded SSc-PH patients and those with extensive ILD on HRCT. Corroborating this hypothesis, an association between peripheral muscle dysfunction and reduced lung function has been previously described (11), and it reinforces the importance of studying the various SSc phenotypes individually.

SSc patients have difficulty performing activities that require physical effort. This situation is at least partly due to vasculopathy, which makes blood flow to the cardiopulmonary system inadequate and, together with musculoskeletal limitations, generates exercise intolerance (34). In the present study, we observed a shorter walking distance in patients than in healthy controls (P=0.006). Unlike most studies, we evaluated the 6MWD relative to ethnicity, age, height, and delta heart rate using predicted values for our population (30). This approach allowed a more reliable analysis of the distance walked when the two groups were compared (15). Notably, when analyzing only the SSc-ILD patient subgroup, we sought to understand the real contribution of ventilatory mechanics to the 6MWT results. We adopted this approach because many non-pulmonary aspects of SSc (including the effects of musculoskeletal conditions) contribute to the test results and decrease the ability to measure changes in lung function (17).

Generally, the correlations between traditional PFTs and the 6MWD are poor, reflecting both the heterogeneity of pulmonary involvement and the absence of an ideal parameter of pulmonary function that can accurately measure pulmonary involvement (5). In the present study, we observed reasonable correlations between the 6MWD and the parameters most commonly used in the follow-up of SSc-ILD (FVC and DLco). In agreement with our results, Deuschle et al. (5) showed a weak correlation between the FVC and 6MWD in patients with limited SSc-ILD on HRCT. Accordingly, a meta-analysis including 43 studies (3,185 patients) (16) evaluated the exercise capacity in patients with SSc with and without vascular or pulmonary involvement. Considering the entire population, the 6MWD was poorly correlated with parameters of traditional PFTs, such as the FVC and DLco. One possible explanation for these poor correlations is the multifactorial nature of the low exercise capacity in SSc patients and a reduced contribution of ILD in cases with limited pulmonary parenchymal involvement (5).

In the quest for construction of a prediction model for the 6MWD in SSc-ILD patients, we observed that the phase III slope_N2SBW_ was the only significant independent variable explaining the walking distance. In our model, 60% of the variability in the 6MWD was explained by the phase III slope_N2SBW_. In SSc-ILD, the probable causes of inhomogeneity in the ventilation distribution measured by the phase III slope_N2SBW_ included ventilation-perfusion mismatching, rapid and shallow breathing, and increased dead-space ventilation due to parenchymal distortion and vascular destruction of the fibrotic lung (35). Since monitoring of SSc-ILD patients through HRCT is unjustifiable due to the constant exposure to radiation, the N_2_SBW test may contribute to stratification and follow-up of these patients in the future, as our results suggested.

Other variables measured during the 6MWT, such as SpO_2_, may be more sensitive to important clinical changes than the distance walked, as previously demonstrated in patients with idiopathic pulmonary fibrosis (36). More recently, Rizzi et al. (17) observed that stratification of SSc-ILD patients based on the degree of desaturation during the 6MWT might be important for interpretation of serial changes. However, the correlations between SpO_2_ and pulmonary and muscle function parameters were weak and nonsignificant in our study. Importantly, the cutaneous manifestations of SSc, in particular the vasospasm caused by RP, can represent significant challenges for the accurate noninvasive measurement of oxygenation during exercise. Thus, our evaluation of oxygen desaturation using a finger probe may have been impaired, since the preferred method for monitoring SpO_2_ in patients with SSc is a forehead probe (17). In fact, Wilsher et al. (37) observed moderate and significant correlations during the 6MWT between oxygen desaturation using a forehead probe (but not a finger probe) and changes in the FVC, DLco, and radiological extent of the disease on HRCT.

We also sought to construct a predictive model for SpO_2_ in the 6MWT in SSc-ILD patients. Differently from the 6MWD model, the SpO_2_ model showed the quadriceps strength and FVC/DLco ratio as significant independent variables with an explanatory capacity of only 26%. In accordance with these findings, Lima et al. (14) found a relationship between quadriceps strength and performance during exercise in SSc patients. Changes in the small blood vessels of the skeletal muscles may influence the cellular oxygen supply and thus contribute to poor performance during exercise (14). This hypothesis is also corroborated by the FVC/DLco ratio, which when greater than 1.6 in SSc patients may reflect microangiopathy and systemic vascular damage extending beyond the pulmonary vasculature (38).

Finally, we did not observe a significant correlation between the 6MWD and mRSS. In contrast, the study by Pugnet et al. (39) showed that the mRSS is an independently associated factor with a lower 6MWD. Since the sample evaluated by Pugnet et al. (39) had a worse mRSS, skin damage might have negatively impacted movement limitations during the 6MWT.

The strength of this study is that it is the first to show the effect of ventilation distribution heterogeneity on the 6MWD in a subgroup of SSc-LD patients and limited lung parenchymal involvement. However, similar to other studies, this study also had some limitations. First, our sample was small, but we were careful to eliminate several confounding factors that could compromise our results, namely patients with SSc-PAH and extensive disease on HRCT. Second, the SpO_2_ measurement could have been performed using means other than a finger probe to minimize interference with detection of the RP oximetry signal. Despite these limitations, we believe that our results provide a perspective for use of the N_2_SBW test in longitudinal studies to verify its prognostic value in SSc-ILD patients.

In conclusion, the present study showed heterogeneity in the ventilation distribution in SSc-ILD patients with limited pulmonary parenchymal involvement and without PH, which is one factor that contributes to a shorter distance walked during the 6MWT. In addition, muscle dysfunction and abnormal lung diffusion at least partly explain the oxygen desaturation of these patients during the 6MWT.
